# What differs between happy and unhappy people?

**DOI:** 10.1186/s40064-016-1929-7

**Published:** 2016-02-29

**Authors:** Ljiljana Kaliterna-Lipovčan, Zvjezdana Prizmić-Larsen

**Affiliations:** Ivo Pilar Institute of Social Sciences, Marulićev trg 19, 10000 Zagreb, Croatia; Washington University in St. Louis, St. Louis, MO USA

**Keywords:** Happiness, International Wellbeing Index, Trust, Leisure

## Abstract

This study explores the determinants (demographic, personal, behavioural, and social) by which happy and unhappy people differ. The primary sample from which the participants were chosen was a representative sample of Croatian citizens (N = 4000). On the basis of the distribution of overall happiness the sample of the highest (the happy group) and the lowest 10 % of participants (the unhappy group) were selected. The happy group (N = 400) represented the upper end of the happiness distribution, while the unhappy group (N = 400) represented the lower end of the distribution. The questionnaire included demographic characteristics (age, gender, income, and education), ratings of subjective health status, satisfaction with specific personal and national domains (IWI-International Wellbeing Index), trust in people, and trust in institutions. Frequency of various leisure activities, and involvement in the community life were also reported. The differences in examined variables were analysed between the two groups. Results showed that the happy individuals were younger, with higher income, and with higher education than unhappy ones. After controlling for age, income, and education level, the happy people were found to be more satisfied with personal and national wellbeing domains, of better subjective health status, reported higher trust in people and institutions, and were more engaged in leisure activities and community life than the unhappy ones.

## Background

The scientific interest in individual’s wellbeing and its impact on society is focusing on examining the predictors of happiness, exploring what are the benefits of being happy, as well as finding the ways to improve wellbeing on individual or national levels. The relationships between wellbeing measures and determinants such as income, health, education, marital status, age, gender, job characteristics have been demonstrated and established (for review see Diener 2013; Diener et al. 2015; Dolan et al. 2008). Research showed that higher level of happiness was associated with better health, higher education, and higher work satisfaction. Although the association between income and happiness is still a matter of scientific debates, in general, studies reveal a positive relationship between wealth and happiness (Graham 2011; Dolan et al. 2008).

Benefits of being happy are documented in the literature (for reviews see Lyubomirsky et al. 2005; Diener 2013; Sirgy 2012). It was reported that happy people function better than unhappy ones in various life domains that De Neve et al. (2013) categorized into (a) health and longevity; (b) income, productivity, and organizational behaviour; and (c) individual and social behaviour. According to authors the experience of high wellbeing encourages individuals to pursue goals that are capacity-building to meet future challenges. At the physiological level, positive emotions have been found to improve immune, cardiovascular, and endocrine functioning, while negative emotions are detrimental to these processes (De Neve et al. 2013). Therefore, feeling happy is not just a pleasant outcome, it also can be predictor and cause of future behavior (Diener 2013) and may have protective role in future health (Steptoe et al. 2015).

While in recent years the increasing number of research examined happy people, the very happy people are not so much in focus of researchers. One of the rare studies on high happiness is that of Diener and Seligman (2002) who compared the very happy people with average and very unhappy ones. In comparison with unhappy people, people who reported high levels of happiness were found to be highly sociable and with stronger social relationships, were more extraverted, more agreeable, less neurotic, and scored lower on several measures of psychopathological symptoms. In addition, they felt pleasant emotions much of the time, felt unpleasant emotions too, but they rarely felt euphoria. Authors concluded that very happy people have a functioning emotion system that can react appropriately to life events (Diener and Seligman 2002). Another study by Oishi et al. (2007) compared moderately happy with very happy people in order to examine the optimal level of happiness. Results showed that optimal levels of happiness differ depending on domains. For example, the highest level of happiness is optimal in the domains of relationships and volunteer work, while moderate level of happiness is optimal for domains of income and education. Authors explained the findings with different motivation associated with the outcomes. For self-improvement motivation the moderate level of happiness is the optimal, as being not completely satisfied leads to achieve more (i.e. get better income or education). On the other side, for the intimate relationships the highest level of happiness is the optimal, as at that level individuals are the most satisfied having no need to change for better.

There is evidence that happy and unhappy individuals differ also in the way in which they behave (Otake et al. 2006), respond to life events, and react in daily situations (Lyubomirsky 2001). Besides the desire to be kind and to be more attuned to the kindnesses, happy people were found to be more likely to behave in kind ways, compared to less happy people (Otake et al. 2006). Examining differences in daily activities in relation to happiness Robinson and Martin (2008) showed that happy people were more active in social activities, religion, reading the newspapers, and less in watching TV than unhappy people. Brajša-Žganec et al. (2011) found that active participation in various leisure activities contributed to better subjective wellbeing. Volunteerism was also found to be related to happiness in a way that people who were happier invest more hours in volunteer work (Thoits and Hewitt 2001), or that people who volunteer are happier than people who do not (Han 2015; Borgonovi 2008). However, some authors did not find any relationship between volunteering and happiness (Haller and Hadler 2006).

Many researchers nowadays examine individual wellbeing in relation to social conditions, and emphasise the importance of individual wellbeing for society (Veenhoven 2009; Diener et al. 2015). When measuring social variables research includes various measures of trust into consideration (Bartolini et al. 2015; Vinson and Ericson 2014). Trust is considered as a multidimensional phenomenon including several aspects of people’s attitudes towards other people, institutions, and society as a whole. Trust in people, and trust in family were found to be positively associated with happiness and life satisfaction (Vinson and Ericson 2014). However, in the study of Leung et al. (2011) conducted in Canada only trust in people within one’s family was found to be related to happiness, while trust in neighbors and strangers was not. The authors explained the findings as a consequence of possible disconnectedness of people within that society. The relationship between trust and subjective wellbeing might be context specific and could be influenced by social, cultural, and political characteristics of the society (Ekici and Koydemir 2014).

## Present study

What makes people happy or unhappy in society they live is important research question. Various correlates of subjective wellbeing have been documented. Happy people tend to function better in different areas of life and report to be more active in the community than unhappy ones. The present study is aimed at exploring the differences between very happy and very unhappy people within society in the set of various personal, social, and behavioural variables.

First, we tested if the happy/unhappy groups differ in demographic variables such as age, gender, income, and educational level. Based on the results of previous studies on the relationship between happiness and demographic variables we hypothesized that happy people would be of younger age, higher income, and with higher education level than unhappy ones, but no gender differences were expected (Dolan et al. 2008; Kaliterna Lipovcan et al. 2007). Demographic variables that we found to differ between happy/unhappy groups were used as covariates in exploring further the differences in other variables.

Second, we explored the differences between happy/unhappy groups in various personal, behavioural, and social measures:

- Personal variables included subjective wellbeing measures covering life satisfaction, satisfaction with personal life domains, and subjective health status. We hypothesized that the existing findings on associations between happiness and other personal variables would be confirmed in direction that happy people would be more satisfied with their life, personal life domains, and of better perceived health status than the unhappy people (Diener 2013).
- Behavioural variables included leisure activities (i.e., engaging in different daily activities in free time) and involvement in the community work. Our hypothesis was that happy people would be more involved in the community work and more engaged in the leisure activities than unhappy ones (Han 2015; Brajša-Žganec et al. 2011).
- Social variables included trust in people, trust in institutions, and satisfaction with various national domains. We predicted that happy people would report higher trust in people and in institutions, and would be more satisfied with national domains than unhappy people (Bartolini et al. 2015; Vinson and Ericson 2014).

## Methods

### Participants

Participants for this study were chosen from the nationally representative sample of Croatian citizens on the basis of their ratings of the overall happiness. The primary sample from which the participants were selected was a representative sample of Croatian citizens (N = 4000) recruited as a part of a public opinion research project. The participants for the primary sample were chosen as a multi-stage probability-based sample of Croatian population. To ensure statistically representative results for the defined target population, 200 sample points were drawn on the basis of the latest statistical data on regional, community, and town levels. Two-stage stratification was used, by region and the size of residence, and addresses were randomly selected at each sampling point. Out of 7.964 contacted persons, 4.000 agreed to participate, so that participation rate was 50.2 %. The representativeness of the sample was checked by comparisons to demographics according to the census of 2001.

For the purpose of this study on the basis of the distribution of overall happiness we selected the subsamples of the highest and the lowest 10 % of participants, total of N = 800. Thus two groups were defined, each of them with N = 400 participants. The happy group represented the upper end of the happiness distribution (M = 10, SD = 0), while the unhappy group represented the lower end of the happiness distribution (M = 2.5, SD = 1.44). Demographic characteristics of selected groups are presented in Table 1.

**Table 1:** Demographics variables for the happy and unhappy groups

Demographics variables	Happy (N = 400)	Unhappy (N = 400)
Age		
Mean (SD)	46 (18.3)	53 (17.2)
Range	18–86 years	18–85 years
Gender		
Women	221 (55 %)	202 (50 %)
Men	179 (45 %)	198 (50 %)
Education		
Elementary (1–8)	98 (24 %)	159 (40 %)
High school (9–12)	219 (55 %)	183 (46 %)
Graduate and higher (>12)	83 (21 %)	58 (14 %)
Monthly income divided by number of persons in family (in Euro)^a^		
<70–139	42 (11 %)	50 (23 %)
140–279	99 (24 %)	145 (37 %)
280–558	169 (43 %)	110 (28 %)
559+	84 (22 %)	58 (12 %)

^a^The income was recalculated in Euros based on currency rate of November 2008 (1 Euro = 7.18 Croatian Kuna)

### Instruments

The range of demographic (age, gender, income, and education), personal (life satisfaction, personal wellbeing, and health status), behavioural (leisure activities and involvement in the community work), and social variables (trust in people, national wellbeing, and trust in institutions) were employed.

#### Demographic variables

The demographic variables included gender, age, education level, and per capita household income (Table 1). Income was defined as a monthly income of the household, divided by number of persons in the household. It was divided into four categories: less than 139 Euro, 140–279 Euro, 280–558 Euro and more than 559 Euro. Education level was measured by three categories: elementary school (1–8 years of schooling), high school (9–12 years of schooling), and graduate and higher (more than 12 years of schooling).

#### Personal variables

##### Happiness

To measure overall happiness we used question “In general, how happy do you feel?” which participants rated on the 11-point scale ranging from 0 as “extremely unhappy” to 10 as “extremely happy”. On the basis of the distribution of the overall happiness we selected the highest and the lowest 10 % of participants. The measure is adapted from the Fordyce Happiness Scale (Fordyce 1988).

##### Life satisfaction

To measure life satisfaction we used question, “All things considered, how satisfied are you with your life as a whole nowadays?” which participants rated on the 11-point scale ranging from 0 as “extremely dissatisfied” to 10 as “extremely satisfied” (Huppert et al. 2009).

##### Personal Wellbeing Index

Wellbeing in specific personal domains was assessed by *Personal Wellbeing Index* (PWI) which is part of the International Wellbeing Index (IWI; Cummins 2002). It measures satisfaction with seven life domains: material status, personal health status, achievement in life, relationships with family and friends, feelings of physical safety, acceptance by the community, and future security, rated on an 11-point rating scale ranging from 0 as “not at all satisfied” to 10 as “extremely satisfied”. The scores are averaged across the domains. The Cronbach alpha for the scale was 0.87.

##### Health

Health status was measured with one item “In general how would you describe your health” on the 5-point scale where 1 means “very poor” and 5 means “excellent”.

#### Behavioural variables

##### Leisure activities

As behavioural variable the frequency of engagement in leisure activities was used. The list consisted of 15 activities that people might engage in during their free time. Subjects had to rate how often they engage in each of the 15 activities on the 8-point scale ranging from 1 as “never” to 8 as “every day”. The scoring was done according to Brajša-Žganec et al. (2011) who identified three groups of leisure activities. The *Visiting cultural activities* consists of 7 items (going to theatre, art exhibition, concert, cinema, daily excursion, doing hobbies, and reading books), *Active socializing and going out activities* consists of 4 items (attending sport events, playing sport, going to restaurant, going to coffee bars), and *Family and home activities* consists of 4 items (visiting friends and relatives; shopping; going to the church; watching TV). The scores were calculated as the average ratings for each group of activities. The reliability analysis showed relatively good properties for scales of *Visiting cultural activities* (α = 0.74), and *Active socializing and going out activities* (α = 0.75), but poor for *Family and home activities* (α = 0.37).

##### Involvement in the community life

The frequency of involvement in the community life was used as an additional behavioural variable. Subjects were asked to indicate how often they have been involved in active helping in the community life (defined as helping to organize community events, to clean the environment, or to be part of civil organization) on the 3-point scale ranging from 1 as “not at all involved” to 3 as “regularly involved”.

#### Social variables

##### National Wellbeing Index

Wellbeing in specific social domains was assessed by *National Wellbeing Index* (NWI) which is part of the International Wellbeing Index (IWI; Cummins 2002). It measures satisfaction with living conditions in the country. Participants have to rate how satisfied they are with: economic situation, state of environment, social conditions, government, business and national security. It was rated on an 11-point rating scale ranging from 0 as “not at all satisfied” to 10 as “extremely satisfied”. The scores are averaged across the domains. The Cronbach alpha for the scale was 0.87.

##### Trust

To measure trust in people we used the question “Would you say that most people could be trusted?” and subject had to rate on the 11-point scale from 0 as “cannot be trusted” to 10 as “could be completely trusted” (Huppert et al. 2009).

##### Trust in institutions

The list of 14 institutions (e.g., government, universities, court, non-governmental associations) was presented and the subjects rated how much they trust each of them on the scale ranging from 1 as “not at all” to 4 as “very much”. In order to reduce and classify items into smaller number of meaningful categories the principal component analysis with varimax rotation was performed on the whole sample of N = 4000 participants. Based on scree plot and the parallel analysis method for determining the number of common factors to retained, three factors were extracted explaining 54 % of total variance. Detailed information on factors’ loadings and communalities are presented in the Table 2. As shown in Table 2, few items exhibited low to moderate communalities in the obtained factor model. Eliminating the variables with lowest communalities increased the variance explained, but did not change the main findings so we decided to accept the structure that comprised all included variables.

**Table 2:** The exploratory factor analysis of trust in the institution scale with factor loadings and communalities (N = 4000)

Items	Factor 1 Trust in public institutions	Factor 2 Trust in government institutions	Factor 3 Trust in non-government institutions	Communality
1. Schools	0.78			0.66
2. Universities	0.75			0.61
3. Military	0.67			0.50
4. Health care	0.64			0.47
5. Police	0.55	0.38		0.45
6. Church	0.52			0.34
7. Parliament		0.81		0.74
8. Government		0.81		0.72
9. The courts		0.61		0.48
10. Political parties		0.57	0.51	0.58
11. Non-government association			0.80	0.67
12. Trade union association			0.77	0.65
13. Media			0.56	0.37
14. President			0.43	0.28

Loadings bellow 0.30 are not presented in the table

The first factor accounted for 35 % of the total variance and it was labelled *Trust in public institutions*. It consisted of 6 items: trust in schools, universities, military, police, health care, and church. The second factor labelled as *Trust in government institutions* accounted for 10 % of total variance. It consisted of 4 items: trust in government, parliament, political parties, and the court. The last factor accounted for 9 % of total variance and it was labelled *Trust in non*-*governmental institutions*. It consisted of 4 items: trust in non-government associations, trade union association, media, and the president. The scores were calculated as the average ratings of trust in each group of institutions. The reliability analyses showed α = 0.79 for *Trust in public institutions*, α = 0.79 for *Trust in government institutions*, α = 0.65 for *Trust in non*-*governmental institutions*.

### Procedure

The survey was conducted in November 2008 by “face to face” interviews in participant’s homes. All interviewers attended training sessions to become familiar with the questionnaire and procedure for selecting survey participants within a household. The respondents were told that responses were anonymous.

### Data analysis

In the present study, exploratory factor analysis was conducted to classify and reduce the number of items for the Trust in the institutions scale (14 items). The Chi square test was used to test the differences in gender, monthly income, and education level between the groups, while the differences in age were tested by *t* test. We used three multiple analysis of covariance (MANCOVA) when assessing for statistical differences on three sets of multiple continuous dependent variables separately (personal, behavioural, and social variables), while controlling for multiple covariates (demographic variables). The Bonferroni corrections were used as adjustment for multiple comparisons. Two measures of effect size were calculated and reported, partial eta-squared (η_p_^2^) and Cohen’s d value. For partial eta square, the suggested norms for a small effect size is 0.01, a medium effect size is 0.06 and a large effect size is 0.14 (Cohen 1988). The commonly used interpretation of d value is to refer to effect size as small (d = 0.2), medium (d = 0.5), and large (d = 0.8) as suggested by Cohen (1988).

All analyses were performed using Statistical Package for the Social Sciences (SPSS) version 21.

## Results

### Demographic variables

We tested if happy and unhappy groups differed in age, gender, income, and level of education. The results showed that happy people were significantly younger (M = 46, SD = 18.3) than unhappy ones (M = 53, SD = 17.2) by almost 7 years (t = 5.42, df = 798, p < 0.001). The groups did not differ in gender (χ^2^ = 1.81, df = 1, n.s.), but they significantly differed in the level of education (χ^2^ = 22.14, df = 2, p < 0.001) with happy group being of higher educational level than the unhappy one. Finally, the groups differed in their income (χ^2^ = 48.42, df = 3, p > 0.001). The happy people reported to have higher income than unhappy ones.

We replicated the previous findings on the associations between the happiness and demographic variables and confirmed the first hypothesis that happy people tend to be younger, with higher income, and of higher education than unhappy ones, but they did not differ significantly in gender.

In further analyses we included age, income, and education level as covariates.

### Personal variables

The results of MANCOVA which tested the differences in the set of personal variables between two groups yielded a significant multivariate main effect for the level of happiness (Wilk’s λ = 0.30, F_3,765_ = 587.13, p < 0.001, η_p_^2^ = 0.70) showing that overall difference among groups was significant. Univariate tests (Table 3) revealed that this effect was significant for the life satisfaction, Personal Wellbeing Index, and health status. The happy group reported higher life satisfaction (d = 2.95), higher satisfaction with personal life domains (d = 2.22), and better subjective health status (d = 1.13). Those findings supported and confirmed the hypothesis on positive relationships between happiness and personal wellbeing variables, happy people were found to perceive their health as better, and they were more satisfied with their life and personal life domains than unhappy people.

**Table 3:** Descriptive statistics of personal, behavioural and social variables for the happy and unhappy groups

Variables (theoretical range)	Happy N = 400	Unhappy N = 400	Statistical test^a^	η_p_^2^
	Mean (SD)	Mean (SD)		
Happiness (0–10*)*	10.0 (0)	2.5 (1.44)		
*Personal variables*				
Life satisfaction (0–10)	8.9 (1.77)	3.2 (2.08)	F_1,767_ = 1455.86***	0.66
Health (1–5)	4.1 (0.97)	2.9 (1.14)	F_1,767_ = 181.23***	0.19
Personal Wellbeing Index (0–10)	8.1 (1.38)	4.5 (1.82)	F_1,767_ = 832.35***	0.52
*Behavioural variables*				
Leisure activities (1–8)				
Visiting cultural events	2.7 (0.97)	2.2 (1.06)	F_1,767_ = 9.80**	0.02
Active socializing and going out	2.9 (1.59)	2.3 (1.42)	F_1,767_ = 2.92 ns	0.00
Family and home activities	5.4 (0.93)	4.9 (1.15)	F_1,767_ = 36.80***	0.05
Involvement in the community (1–3)	1.4 (0.64)	1.2 (0.53)	F_1, 767_ = 3.64*	0.01
*Social variables*				
National Wellbeing Index (0–10)	5.4 (2.08)	3.6 (1.71)	F_1,769_ = 155.52***	0.17
Trust in people (0–10)	5.0 (2.59)	3.5 (2.09)	F_1,769_ = 75.67***	0.09
*Trust in the institutions* (1–4)				
Government institutions	1.9 (0.67)	1.7 (0.63)	F_1,769_ = 19.33***	0.03
Public institutions	2.5 (0.66)	2.2 (0.66)	F_1,769_ = 51.13***	0.06
Non-government institutions	2.3 (0.65)	2.1 (0.67)	F_1,769_ = 19.18***	0.02

*ns* non-significant

* *p* < 0.05; ** *p* < 0.01; *** *p* < 0.001

^a^Three MACNOVAs were performed for personal, behavioural, and social variables separately to test the differences between happy and unhappy groups. The overall Wilks’s lambda for each MANCOVA was significant, *p* < 0.001. The tests of significance for individual dependent variables are presented. In all analyses the covariates were age, income and education level; η_p_^2^ = Partial eta-squared, effect size measure

### Behavioural variables

The results of MANCOVA which tested the differences in frequency of leisure activities and the involvement in the community life between the two groups yielded a significant multivariate main effect (Wilk’s λ = 0.95, F_4,764_ = 10.58, p < 0.001, η_p_^2^ = 0.05) showing that overall difference among groups was significant. Univariate tests (Table 3) revealed that the happy group reported to spend more time in activities listed in the scales of Visiting cultural events, and Family and home activities than the unhappy group. No differences were found in time spent in Active socializing and going out activities. Additionally, the happy group did differ from the unhappy one in the involvement in the community life. Those results confirmed our hypothesis that happy people would be more involved in the community work (d = 0.34) and more engaged in leisure activities than unhappy ones (visiting cultural events d = 0.49; family and home activities d = 0.48). However, one component of the leisure activities that concerned the engagement in the social activities did not differ between two groups.

### Social variables

The results of MANCOVA which tested the differences in the set of social variables between the two groups yielded a significant multivariate main effect (Wilk’s λ = 0.79, F_5,765_ = 39.73, p < 0.001, η_p_^2^ = 0.21) showing that overall difference among groups was significant. Univariate tests revealed that this effect was significant for National Wellbeing Index, trust in people, and trust in institutions (see Table 3). The happy group reported higher satisfaction with national domains (d = 0.94), higher trust in people (d = 0.64) and in institutions (government d = 0.31; public d = 0.45; non-government d = 0.30). Those findings supported the hypothesis that happy people would report higher trust in people, trust in institutions, and better national wellbeing than unhappy people.

Descriptive statistics for three sets of variables (personal, behavioural, and social), results of MANCOVAs and partial eta-squared values for the happy and unhappy groups are presented in Table 3.

## Discussion

The goal of the present study was to explore what are the differences between the very happy and very unhappy people. Happiness in our study was defined as overall, general level of happiness rather than momentary mood or state. Three sets of dependent variables were used in our analyses: personal, behavioural, and social variables. In all three variable sets the significant differences between the happy and unhappy citizens were found.

The differences between the happy and unhappy individuals in demographic variables follow the well-established findings in the literature and confirm our first hypothesis. We found that the happy Croatian citizens were 7 years younger, more educated, and with higher income than unhappy ones. No gender differences were established. Those findings so far are in line with the previous studies conducted in Croatia, showing that wellbeing is higher when the person is younger (Kaliterna Lipovcan and Prizmic Larsen 2007) and with higher income (Kaliterna Lipovcan et al. 2007).

While controlling for demographic variables (age, income, and educational level) the happy citizens rated themselves as of better health status, more satisfied with their life as a whole, and more satisfied with various personal life domains than the unhappy ones. Since the happiness, life satisfaction, and satisfaction with different life domains are measures of a broader concept of subjective wellbeing, the positive associations between them were expected. These results can be interpreted as showing the concordance between affective (happiness) and cognitive (satisfaction with life and life domains) measures of subjective wellbeing. The finding that the happy people perceived their health status as better than unhappy ones is in accordance with previous findings on subjective wellbeing and health (Diener and Chan 2011). In an extensive analysis of Eurostat and European Value Survey data Gataulinas and Bancevica (2014) explained the interrelation between subjective health and subjective wellbeing suggesting that the health affects the overall level of wellbeing through its role in satisfaction of needs. Good health creates prerequisites to satisfy needs in a full extent, while disability and disease creates an obstacle to satisfy human needs and in that sense it can reduce satisfaction with different life domains and with life as a whole.

Concerning the behavioural variables, the prediction that happy and unhappy groups would differ in participation in leisure activities was partly confirmed. We found that the happy people reported to engage more frequently in activities concerning family and home (such as visiting friends and relatives, shopping, going to the church, and watching TV), as well as in visiting various cultural events (i.e., going to theatre, art exhibition, concert, cinema, doing hobbies, and reading books) than do unhappy people. This result is in accordance with previous findings about positive relationships between leisure activities and subjective wellbeing (Brajša-Žganec et al. 2011; Newman et al. 2014). In the bottom-up theoretical model of Newman et al. (2014) authors argue that engaging in leisure could potentially promote various dimensions of subjective wellbeing in the leisure domain, which subsequently promotes global wellbeing. However, there is also a possibility of the reverse direction. The level of happiness may have an effect on leisure activities in the way that happy people would be more likely to involve in leisure activities than unhappy ones. However, because of cross-sectional design of our study this assumption is only speculative. Interestingly, the differences between our two groups in engaging in activities of socializing and going out were not found significant, what is not in accordance with other research showing that socializing is an important component of happiness and that happy people socialize more than unhappy ones (Robinson and Martin 2008). One possible explanation of this result could be that some of the activities listed in the scale, such as going to café, are quite common activities in Croatia that almost everybody is involved in it, while other, such are dining in restaurants or playing and attending sport events are quite rare. Therefore, the frequency of involvement in these activities does not differentiate by level of happiness or preferences, but rather are part of daily living.

The involvement in the community work also differed between happy and unhappy citizens, but it should be pointed out that although significant, the difference between the two groups yielded small effect size. Happy citizens reported to being more frequently involved in helping to organize community events, to clean environment and to actively participate in civil organizations than unhappy ones. The involvement in the community work was quite low in both groups, which is in line with the findings that participation in voluntary associations is low across Europe (Wallace and Pichler 2009), and especially low in Croatia (Eurofound 2014). One of the reasons that Croatian citizens do not participate more in social activities is that they perceive that as individuals they cannot influence the decisions affecting local or more global community as documented by Franc et al. (2012) in a national survey.

Trust in people, trust in institutions and satisfaction with various national life domains were considered in these analyses as measures of social variables or in a broader sense social capital, described as “networks, together with shared norms, values and understandings which facilitate cooperation within or among groups” (OECD 2001, p. 41). Predicted findings on association between happiness and social variables were confirmed in our study. The happy people reported higher trust in people and in institutions (government, non-government and public), and were more satisfied with various national domains than unhappy ones. This is in line with other studies showing positive associations between social capital and wellbeing (Bartolini et al. 2015; Yetim and Yetim 2014; Guven 2011). Examining the causal relationship between self-reported happiness and measures of social capital Guven (2011) found that happiness induces a higher level of trust to others. Happier people were found to have higher respect for law and order, help others more, have more memberships, and have a higher desire to vote. His study also showed that happier people perform more volunteer work, are more attached to their neighborhoods, and participate more in community events, social gatherings, cultural events, local politics, and religious events. Author speculates that happy people can be viewed as better citizens (Guven 2011). The results of our study support this speculation, since it was found that, besides having higher personal wellbeing, what is important from the individual’s point of view, happy people are more active in their everyday life, and function better in society than unhappy people.

## Conclusions

What differs between happy and unhappy people? Our results showed that almost everything that we compared differed between the groups of very happy and very unhappy Croatian citizens. Very happy citizens were found to be 7 years younger, better educated and with higher income. They reported to be of better health, more satisfied with their life as a whole, and more satisfied with various personal life domains than the unhappy ones. Also, the happy people were more involved in community work and engaged in various leisure activities, except the activities associated with socializing and going out. Finally, the happy people showed higher trust in people, trust in institution, and better national wellbeing than unhappy people. Our results confirmed the findings that being happy have benefits for individual as well as for the society (Diener 2013; Steptoe et al. 2015). Examining the characteristics of very happy versus very unhappy people in one’s society could be useful tool for specifying domains where public policies should be implemented in order to improve the wellbeing of the society.

This study adds to an emerging pool of evidence regarding associations between subjective wellbeing and various life domains. Instead of focusing on specific domain, we used wide range of personal, social and behavioural variables to test the differences between the extreme happy and extreme unhappy people. One of the strength of this study is the usage of nationally representative probability sample from which extreme groups were chosen, so that the participants represent the most happy and most unhappy citizens. In that respect, the established differences point to the domains which should be improved in the society in order to have higher wellbeing of the population.

However, our study has also some weaknesses. The present findings are based on cross-sectional and correlational data that limit inferences about processes and causality, so longitudinal methods would be desirable in exploring causality of those relationships. Another weakness is that all measures were based on self-reports and are potentially subject of measurement biases such are response set bias, memory bias or bias related to mood, as reviewed by Sirgy (2012, pp 574–581). Including measures such are depression or social desirability in further research, could address the problem of response bias toward the negativity or social desirability bias in wellbeing research (Heintzelman et al. 2015). Our findings are limited especially for the set of “behavioural variables” as we did not measured real behaviour, but reports of past behaviour. In the future, the objective measures should be collected additionally, for example, health status obtained from the doctor visits, behavioural measures in the form of the attendance at the community work or activities, or peer reports on the daily activities and various behavioural acts. One of the methodological problems of our study is rather weak explanatory power of scales representing Trust in institutions and Leisure activities, as has been shown by their factor structures and low reliability of one of the Leisure activities scale. The explanatory power of some of those variables might be better represented by the individual variable than as a part of the scale. However, the main aim of our study was not to examine in depth each of the domains studied (personal, behavioural, social), but to show the variety of differences between very happy and very unhappy people. For in depth analyses of particular domain parallel use of objective and subjective measures would help to better explore the associations with wellbeing, as also suggested by Lloyd and Auld (2002) for the domain of leisure activities.
